# Sieve analysis to understand how SARS-CoV-2 diversity can impact vaccine protection

**DOI:** 10.1371/journal.ppat.1009406

**Published:** 2021-03-25

**Authors:** Morgane Rolland, Peter B. Gilbert

**Affiliations:** 1 U.S. Military HIV Research Program, Walter Reed Army Institute of Research, Silver Spring, Maryland, United States of America; 2 Emerging Infectious Diseases Branch, Walter Reed Army Institute of Research, Silver Spring, Maryland, United States of America; 3 Henry M. Jackson Foundation for the Advancement of Military Medicine, Bethesda, Maryland, United States of America; 4 Vaccine and Infectious Disease and Public Health Sciences Divisions, Fred Hutchinson Cancer Research Center, Seattle, Washington, United States of America; 5 Department of Biostatistics, University of Washington, Seattle, Washington, United States of America; Mount Sinai School of Medicine, UNITED STATES

## Summary

Sieve analyses will compare Severe Acute Respiratory Syndrome Coronavirus 2 (SARS-CoV-2) sequences from breakthrough infections in vaccine recipients to the sequences found in placebo recipients to understand if vaccination fails to protect against specific genetic variants, characterize the potential impact of vaccines on SARS-CoV-2 evolution, and help design next-generation vaccines.

### What is sieve analysis?

Initial efficacy trials of vaccines against Severe Acute Respiratory Syndrome Coronavirus 2 (SARS-CoV-2) have shown remarkable vaccine efficacy [1]. One way to understand the mechanism(s) behind vaccine protection is to analyze whether the viruses found in vaccine recipients who became infected differed from the viruses found in placebo recipients. This analysis framework, which compares viral sequences in the vaccine and placebo groups, has been described as sieve analysis. A series of genetic and statistical approaches are used to measure the dissimilarity between sequences isolated from trial participants and the vaccine insert sequence(s), and then these dissimilarities are compared between vaccine and placebo recipients. The genetic consequences of vaccine-induced immune responses can be imprinted in breakthrough infections with differences at the genome, gene, or residue level. In the Human Immunodeficiency Virus 1 (HIV-1) vaccine field, we showed that, despite limited efficacy to prevent HIV-1 acquisition, vaccine recipients who acquired HIV-1 infection had genetic signatures in their breakthrough viruses [2,3]. Beyond comparing sequence variation for the study endpoint of pathogen infection, sieve analysis can compare sequence variation for study endpoints corresponding to symptomatic or severe disease, which are primary endpoints of SARS-CoV-2 vaccine efficacy trials. Sieve analyses focusing on clinical disease endpoints were previously described for clinical malaria [4] and symptomatic virologically confirmed dengue [5]. In addition to developing insights into mechanisms of vaccine protection, an overarching objective of sieve analysis is to understand how viral variants influence the level of vaccine efficacy toward defining a sequence-based biomarker of the virus that predicts how well a vaccine prevents infection or disease with that specific virus. The main public health application of these objectives is to optimize strain selection into the formulation of future versions of vaccines.

### Why is sieve analysis needed for SARS-CoV-2?

SARS-CoV-2 sieve analysis focusing on the primary trial endpoint of symptomatic or severe Coronavirus Disease 2019 (COVID-19) relies on a sufficient number of symptomatic cases to enable robust statistical analysis. The small numbers of vaccine failures for the mRNA vaccines approved for emergency use (with upwards of 90% vaccine efficacy) imply few symptomatic cases, thereby limiting statistical power of sieve analysis. In contrast, the lower estimates of vaccine efficacy against symptomatic infection (in the range of 50% to 75%) reported for some other vaccines indicate that the number of breakthrough cases will not be a significant problem. Yet, to our knowledge, all efficacy trials showed high vaccine efficacy against severe COVID-19, such that so far sieve analysis does not appear possible for the severe COVID-19 endpoint (due to low statistical power).

Beyond the primary endpoint of symptomatic COVID-19, sieve analyses that focus on SARS-CoV-2 infections will be particularly relevant to characterize the effect of viral variation on vaccine efficacy. Since many infections remain asymptomatic, the emphasis on symptomatic COVID-19 means that the vaccine could show excellent (trial defined) efficacy without blocking all SARS-CoV-2 infections. Current trials typically study vaccine efficacy against SARS-CoV-2 seroconversion at 3 to 6 monthly visits but can miss many infections because of waning nucleoprotein antibody detectability and limited RNA PCR nasal swab testing [6]. Therefore, it would be valuable for some vaccine efficacy trials to implement strategies to frequently test trial participants for SARS-CoV-2 infections and to sequence infections. Providing trial participants with home kits could enable frequent testing (e.g., weekly) as was demonstrated in a study where participants self-collected nasal swabs daily for 14 days [7]. First, frequent testing strategies would clarify whether vaccines substantially reduce SARS-CoV-2 transmission at the population level. Preliminary findings suggest that vaccines do not block transmissions to the same extent as they prevent symptomatic disease [8]. Second, frequent screening for asymptomatic infections would allow to study how the protective efficacy of the vaccine against nasal carriage or asymptomatic infection depends on SARS-CoV-2 genetics. The recent spread of outlier variants [9–11] emphasizes the need to rapidly track the impact of vaccine-induced pressure on SARS-CoV-2 evolution.

A structured framework in double-blind or observer-blind randomized trials will provide the most insightful and robust tests for identifying a vaccine-specific effect. Nonetheless, outside of randomized trials, the large-scale distribution of vaccines will likely allow investigators to establish studies to compare infections from vaccinated and unvaccinated individuals in a local setting. Various nonrandomized study designs may be useful for learning about sequence-specific vaccine efficacy. For example, the test negative design—an observational study design where based on symptom-triggered testing those who test positive are cases and those who test negative are controls—is expected to be used widely for postapproval effectiveness studies of SARS-CoV-2 vaccines. These designs have been applied for assessing flu vaccine efficacy against influenza-like illness with different flu strains [12] and can be readily adapted for sieve analysis to assess sequence-specific SARS-CoV-2 vaccine efficacy against symptomatic infection.

### SARS-CoV-2 sieve analysis: How viruses found in vaccine and placebo recipients may differ?

**Fig 1:**
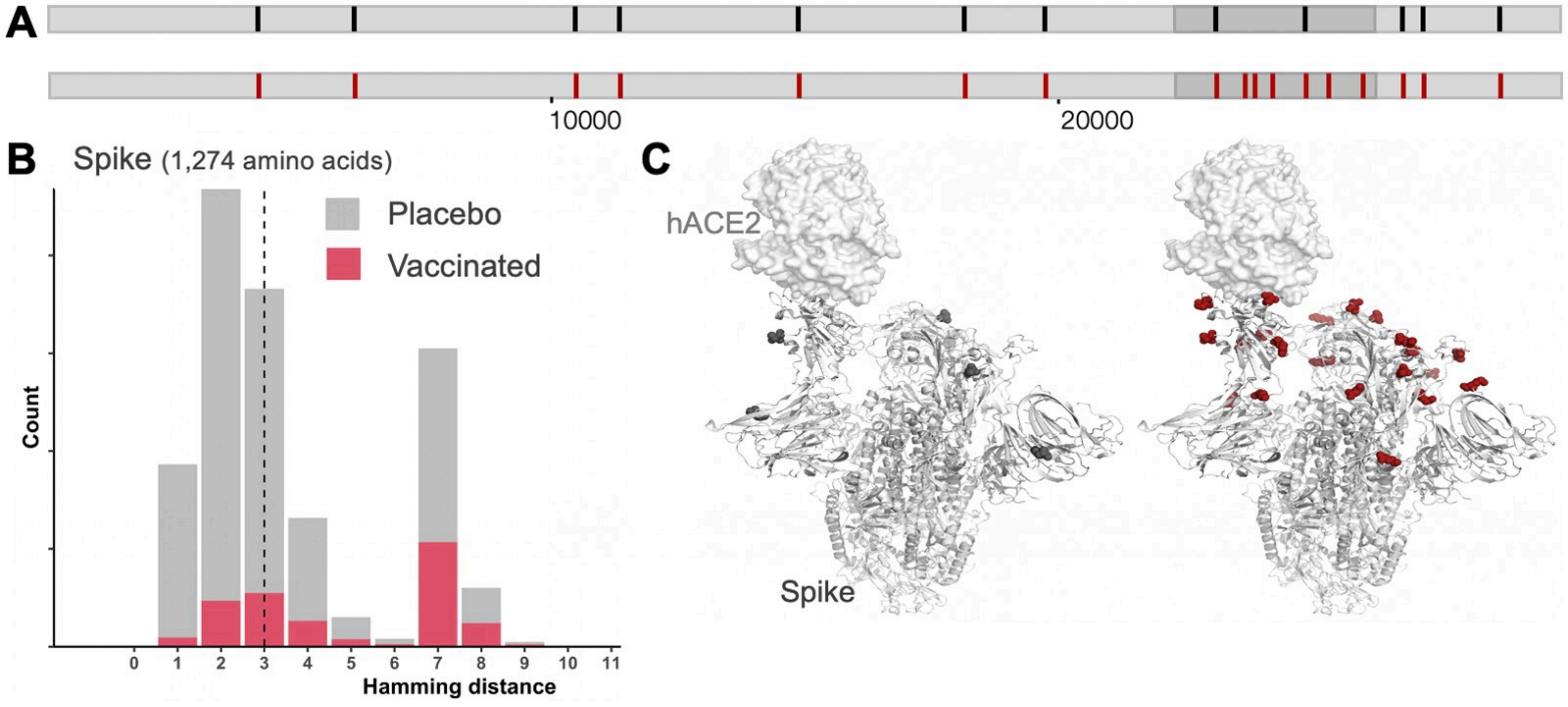
Schematic representation of sieve effects in SARS-CoV-2 sequences from vaccine (red) and placebo (black) recipients who acquired infection in a preventive vaccine efficacy trial. (A) Mutations/vaccine-mismatched residues (represented with tick marks) are preferentially found in vaccine recipients in the gene that is a component of the vaccine, the *spike*. (B) Viruses found in the vaccine group (red) are more distant from the vaccine sequence (Wuhan Hu-1 reference) than viruses in the placebo group (gray). The distribution in gray shows hamming distances between the vaccine and 156,260 circulating Spike sequences sampled between November 1, 2020 and February 1, 2021 [16]. The hamming distance corresponds to the number of differences between the vaccine insert and any circulating Spike sequence (*n =* 1,274 amino acids) (46 sequences showed more than 10 amino acid mutations when compared to the vaccine insert and were not represented). The distribution in gray represents the expected distribution in the placebo group, while the distribution in red represents the viruses infecting vaccine participants (after vaccine sieving). Since the vaccine presents the Spike to the immune system of vaccinated individuals, the distribution of hamming distances was restricted to Spike protein sequences to focus on sites relevant to the specificity of vaccine-induced immune responses. (C) Viruses in the vaccine group showed more vaccine-mismatched residues at contact sites for Spike-specific antibodies including RBD-specific antibodies (shown as red spheres) than viruses in the placebo group (black spheres) due to the presence of vaccine-elicited Spike-specific antibodies in vaccine recipients.

For SARS-CoV-2 vaccines, we hypothesize that vaccines could preferentially block viruses that were the most genetically similar to the vaccine insert. Here, we consider the *spike*, the viral gene sequence used in almost all vaccine candidates, including some that have been granted emergency use authorization such as the Pfizer BNT162b2 and Moderna mRNA-1273 vaccines. These vaccines were based on the first SARS-CoV-2 sequence available, Wuhan-Hu-1 [GISAID (Global initiative on sharing all influenza data) accession EPI_ISL_402125], which was published on January 9, 2020. Comparison of SARS-CoV-2 genomes sampled from vaccine and placebo recipients would show differences between vaccine and placebo groups (Fig 1A). Increased vaccine-blockage of acquisition of vaccine-like viruses would lead to viruses in the vaccine group (breakthrough infections) being more genetically distant from the vaccine insert, here the Spike, than viruses in the placebo group (Fig 1B). A sieving effect could also be manifested by differences in specific determinants of vaccine-induced immunity, e.g., in an antibody or T cell epitope.

Since SARS-CoV-2 vaccine protection will likely be linked to antibody responses targeting the Spike protein and, specifically, the Receptor Binding Domain (RBD), an expected sieve effect is that mutations that were previously identified as associated with RBD antibody neutralization resistance or viral escape would be overrepresented in the vaccine group [13,14] (Fig 1C). Specifically, in vitro studies identified mutations engendered through passage in the presence of RBD-specific antibodies [14] and other mutations that affect the binding affinity to the functional receptor for the Spike, the Angiotensin-Converting Enzyme 2 (ACE2) [13]. Using existing escape and binding affinity maps, we can define hypothetical sieve effects and then test whether these amino acid features are critical via sieve analysis and immunology assays. Sieve analysis could also reveal novel sites of vulnerability as vaccine effects (protection or disease attenuation at the individual level and transmission reduction at the population level) could be mediated by other responses in addition to neutralizing antibodies. Analysis of breakthrough infections could allow the discovery of possible signatures linked to antibody-mediated Fc effector functions or HLA-associated polymorphisms linked to T cell responses.

**Fig 2:**
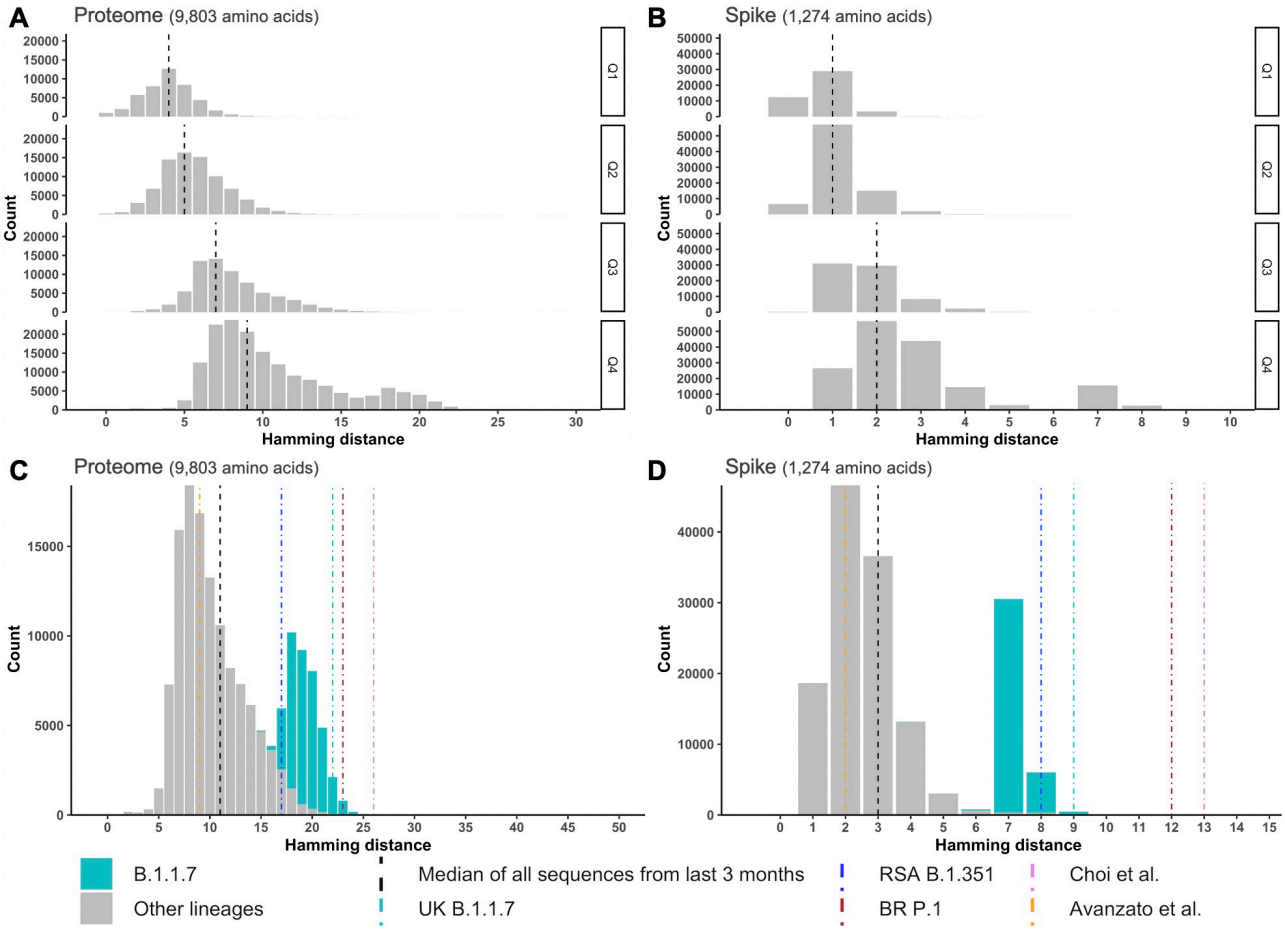
SARS-CoV-2 evolution. (A) The distributions in gray shows hamming distances between the vaccine (Wuhan-Hu-1 reference) and 360,382 circulating sequences separately for each trimester of 2020 (22 sequences from 2019 were added to the set of sequences from the first trimester of 2020) [16]. The number of mutations increased over time with a median of 9 amino acid differences from the Wuhan-Hu-1 reference across the proteome (A) and a median of 2 differences in the Spike (B) at the end of 2020. (C, D) Comparison of circulating sequences to emerging variants of concern. Distribution of distances between the vaccine sequence (Wuhan Hu-1 reference) and circulating sequences sampled between November 1, 2020 and February 1, 2021. The gray distributions represent the diversity across circulating sequences corresponding to SARS-CoV-2 proteomes and Spike. The bimodal distribution is linked to the predominance of B.1.1.7 sequences in the UK (*n =* 36,512 of 156,260 sequences, shown in green). The distance corresponding to outlier variants is represented with colored lines. Sequences from immunocompromised individuals are represented with pink [19] and orange [18] lines. Panels (C) and (D) show that sequences corresponding to the variants of concern B.1.1.7 (originally identified in the UK, shown in green), B.1.351 (originally identified in South Africa, shown in blue), and P.1 (originally identified in Brazil, shown in purple) are very different from previously circulating viruses.

Compared to other RNA viruses, SARS-CoV-2 shows a more limited propensity to mutate [15]. This presents specific challenges and opportunities for sieve analysis. The comparison of sequences sampled up to December 31, 2020 (*n =* 360,382 deduplicated sequences from 137 countries with 44% of sequences sampled in the United Kingdom (UK), downloaded from GISAID on February 1, 2021 [16]) to the Wuhan-Hu-1 reference sequence (the basis for current vaccines) shows the slow accumulation of mutations globally (Fig 2A and 2B). Mutations happen in a stepwise fashion, and, at the end of 2020, circulating sequences showed a median of 9 amino acid mutations when compared to the Wuhan-Hu-1 reference (Fig 2A) and a median of 2 amino acid mutations in the vaccine-targeted Spike (Fig 2B). One of the 2 mutations in the Spike is D614G which is now almost universal. The small number of Spike mutations visible at this time in the pandemic indicates that vaccines should be efficacious against the predominant variants currently circulating. One challenge for sieve analysis is that the difference between vaccine and placebo groups might currently be small. Obtaining deep-sequencing data which would enable to evaluate the presence of mutations found at a low level among sequences from an infected individual may help delineate a small effect. One advantage is that distinguishing sieve acquisition and postinfection effects should be possible for SARS-CoV-2. For more diverse pathogens (e.g., HIV-1), differentiating sieve effects linked to virus acquisition (what virus was transmitted?) versus viral evolution postinfection (did this virus evolve posttransmission?) is poorly tractable. This distinction will be facilitated for SARS-CoV-2 given the limited duration of viral replication in most individuals (less than a month) and the slow diversification process of SARS-CoV-2: One nucleotide substitution is expected to occur in *spike* about every 3 months (given a substitution rate of 0.001 substitution/site/year [17]) (one substitution is observed about every other week across circulating genomes).

### Will SARS-CoV-2 viruses adapt to vaccines?

As part of postlicensure surveillance, sieve analyses would allow description of evolutionary patterns that will be key to infer the potential adaptation of SARS-CoV-2 to vaccines. Characterizing breakthrough infections in current vaccine efficacy trials is a critical tool to identify mutations associated with vaccine failure and to predict the possible consequences of viral adaptation for future vaccine efficacy. While SARS-CoV-2 diversity is still limited globally, the recent emergence of variants with a larger number of mutations than expected raises concerns (Fig 2C and 2D). Fig 2C and 2D shows the distance between the vaccine reference and sequences sampled between November 1, 2020 and February 1, 2021. Comparing this distribution to the one corresponding to the last trimester of 2020 (Fig 2A and 2B) shows that the viral population could rapidly shift; here the bimodal distribution reflects the large proportion of sequences sampled from the UK (24%) where the divergent B.1.1.7 variant quickly became prevalent. In the fall of 2020, the identification of Spike sequences with 5 to 12 mutations in 2 immunocompromised individuals followed for 3 to 5 months [18,19] showed the need for increased surveillance of individuals with persistent infections or those treated with monoclonal antibodies. At the end of 2020, additional viruses with divergent Spike sequences emerged and, more importantly, they were able to spread rapidly in the population [9–11]. The variants B.1.1.7 (originally identified in the UK), B.1.351 (originally identified in South Africa), and P.1 (originally identified in Brazil) have more mutations than what was expected at this time in the pandemic, and a large fraction of these mutations are in the Spike, indicating likely selection pressure behind their emergence.

These findings emphasize the need for reinforcing SARS-CoV-2 sequencing efforts to track its evolution as the pandemic unfolds. Increased vigilance is needed in the months until most of the population has been vaccinated. The selective pressure exerted by the vaccine together with limited vaccine coverage in the population has the potential to open ecological niches where rare variants with potentially unfavorable resistance profiles could outcompete circulating viruses. Preliminary results from the Novavax NVX-CoV2373 vaccine trials conducted in the UK (Phase III) and South Africa (Phase IIb) suggest that such a scenario could occur. Indeed, while the vaccine efficacy was 89.3% in the UK where half of the symptomatic infections corresponded to the B.1.1.7 variant (32 of 60 sequenced cases), vaccine efficacy was 60% in South Africa (among the HIV–negative population) where most symptomatic infections corresponded to the B.1.351 variant (25 of 27 sequenced cases) (https://ir.novavax.com/news-releases/news-release-details/novavax-covid-19-vaccine-demonstrates-893-efficacy-uk-phase-3). This difference in vaccine efficacy parallels a difference in neutralization sensitivity when sera from vaccine recipients are tested against the B.1.1.7 and B.1.351 variants. While the B.1.1.7 variant is neutralized almost as efficiently as the consensus D614G variant (sera from Pfizer, Moderna, and Novavax vaccine recipients), neutralization is significantly reduced against the B.1.351 variant (sera from Pfizer and Moderna vaccine recipients) [20,21]. The decrease in neutralization is likely due to the presence of the E484K escape mutation in the B.1.351 variant (also found in P.1). Importantly, the E484K has recently emerged in the B.1.1.7 background and was found in multiple individuals [22]. Such evidence of convergent evolution emphasizes that the E484K mutation provides a pathway to escape antibody recognition. The E484K mutation together with N501Y and mutations in the NTD supersite that are shared in the emerging variants of concern are evocative of the rapid adaptive evolution potential of SARS-CoV-2. The pressure that vaccination will exert on the virus to adapt coupled with the very large number of infected individuals suggest that the period before widespread vaccination could give rise to multimutations jumps in the SARS-CoV-2 diversification process (that had, so far, been gradual). This could give rise to strains with different properties and maybe to distinct serotypes. Nonetheless, the polyclonal neutralizing response elicited via vaccination likely targets multiple epitopes on the Spike, thereby limiting pathways for viral escape.

In summary, continuous surveillance of SARS-CoV-2 infections is needed and sieve analysis by evaluating breakthrough infections in the context of randomized vaccine efficacy trials, either double-blind or observer-blind, will provide a better understanding of the impact of vaccines on SARS-CoV-2 viruses and their evolution with the possibility of integrating genomic, clinical, and epidemiologic information to the characterization of immune responses elicited by the vaccine. Tracking SARS-CoV-2 evolution as vaccination campaigns are rolled out will be critical to designing next-generation vaccine candidates.
